# Influence of three times weekly alfalfa supplementation on the behavior of beef cows grazing dormant Montana rangeland^1^

**DOI:** 10.1093/tas/txaa098

**Published:** 2020-12-22

**Authors:** Noah Gene Davis, Samuel Aaron Wyffels, Carla Sanford, Timothy DelCurto

**Affiliations:** 1 Department of Animal and Range Sciences, Montana State University, Bozeman, MT; 2 Northern Agricultural Research Center, Montana State University, Havre, MT

## INTRODUCTION

Supplementation is a popular management strategy among livestock producers in western North America. Conventionally, supplementation has been used to bridge the gap between livestock nutrient requirements and the nutrient content of low-quality forage. More recently, there has been interest in using supplementation as a tool to improve livestock distribution on heterogeneous landscapes (Bohnert and Stephenson, 2016; Bailey et al., 2019).

Protein supplements are often the best choice for use with low-quality forages because they improve digestion and increase basal forage intake (Moore et al., 1999; DelCurto et al., 2000). Though commonly fed daily, hand-fed protein supplements can be delivered as infrequently as once weekly to decrease labor costs while maintaining the same level of performance (Kunkle et al., 2000; Klein et al., 2014).

While there is information describing the influence of supplementation on cow behavior (Bailey, 2004; Bohnert and Stephenson, 2016), there is little information describing the behavior of cattle supplemented infrequently (Brandyberry et al., 1992; Schauer et al., 2005), or comparing behavior between days cattle were supplemented and those they were not (Dunn et al., 1988; Sprinkle et al., 2019).

Reducing the frequency of protein supplementation has no effect on performance, however, it may alter grazing behavior. Many livestock producers strive to maximize use of the basal forage resource in large rangeland pastures. So, it is important to understand how infrequent supplementation influences grazing behavior. Therefore, the objective of this research is to evaluate differences in grazing behavior of cows supplemented three times weekly on supplemented vs. non-supplemented days. We hypothesize that cattle grazing behavior is influenced by timing/day of supplementation.

## MATERIALS AND METHODS

The care and use of cattle in this study was approved by the Institutional Animal Care and Use Committee of Montana State University (ACUP #2018-AA14).

This study was conducted in a 645-ha pasture at Montana State University’s Red Bluff Research Ranch in Norris, MT (45°35′N, 111°38′W). Mean annual precipitation is 406 mm, 60% of which comes during the growing season (May through September). Topography of the pasture is a combination of gently sloping alluvial fans, steep hillslopes, and broad ridges. Pasture elevation ranges from 1,415 to 1,715 m. The vegetation is primarily a grassland with an open woodland of Rocky Mountain juniper (*Juniperus scopulorum*) and limber pine (*Pinus flexilis*) on the hillslopes. Dominant herbaceous species are bluebunch wheatgrass (*Pseudoroegneria spicata*), Idaho fescue (*Festuca idahoensis*), needle-and-thread (*Hesperostipa comata*) and isolated patches of cheatgrass (*Bromus tectorum*).

Over two winters, a herd of commercial, March-calving, multiparous Angus cows (*n* = 139 in year 1 and 143 in year 2) grazed in the pasture for 56 d each year (December 14, 2018 to February 8, 2019 and December 12, 2019 to February 6, 2020). The stocking rate was considered light to moderate for the area (2.63 ha∙AUM^−1^). On days 0 and 56, all cows were weighed and ranked by body condition score (BCS; 1–9 scale; NRC, 2016) following a 16-h shrink (Table 1). At 1300 h every Monday, Wednesday, and Friday, all cows were gathered and taken to a central location in the upland region of the pasture. All cows reached the supplement site, between 1400 and 1500 h, where 3.18 kg∙hd^−1^ of alfalfa pellets (17% CP) were delivered on the ground via a truck-mounted cake feeder.

**Table 1.** Forage availability, weather conditions, and cow performance for beef cows grazing between December and February over 2 yr in Norris, MT

	Year 1	Year 2
Standing crop, kg∙ha^−1^	1425.00	592.00
Temperature, °C		
Mean	−3.12	−1.32
Min	−25.60	−16.90
Wind speed, m∙s^−1^		
Mean	4.75	6.30
Max	16.60	20.10
Snow depth, cm		
Mean	5.70	2.97
Max	35.60	20.30
Bodyweight, kg		
Initial	599.00	604.00
Final	602.00	599.00
Body condition score, 1–9^*^		
Initial	5.00	5.40
Final	5.00	5.10

*NRC (2016).

Each year, 18 cows were randomly assigned a global positioning system (GPS) collar (LiteTrack 420; Lotek Wireless, Newmarket, Ontario, Canada). Each collar contained a GPS receiver and 3-axis accelerometer. Collars were programmed to take GPS location readings at 5-min intervals and accelerometer readings at 1-min intervals for the entirety of the study. Distance traveled and rate of travel were estimated between successive points for each location reading (Ganskopp, 2001).

In year 1, each collared cow was observed for 3 ± 1 h and its dominant activity was recorded every minute. Grazing, resting, and traveling made up over 99% of observations, therefore, these were the only activities considered. A behavior prediction model was developed via a random decision forest using the randomForest package in R to estimate grazing and resting time (Liaw and Wiener, 2002; R Core Team, 2019). The dependent variable was activity and the independent variables were the X, Y, and Z axes from the accelerometer data and the rate of travel from the location data. To validate accuracy, only 80% of the observations were used for training the model. The model was then used to predict activity of the remaining 20% of the observations using the location and accelerometer data. The actual and predicted activities were compared, resulting in a model accuracy of 88.7%.

Activity was classified relative to the timing of supplementation. All activity 24 h after gathering the cows for supplementation was considered “post-supplementation”. All activity 24 h prior to gathering the cows for supplementation was considered “pre-supplementation”. Hereafter, this classification will be referred to as the “24-h period”. Data from 1300 h Saturday to 1259 h Sunday does not fall into either category and was excluded from analysis. We developed two datasets from our data. The first summed distance traveled, grazing time, and resting time for each cow over each 24-h period of the study. The second summed distance traveled, grazing time, and resting time for each cow over each hour of the study.

Temperature and wind speed readings were collected on a 5-min interval using a HOBO U30 weather station (Onset, Bourne, MA) deployed in a central location in the study pasture (Table 1). Snow depth was manually measured and recorded daily (Table 1). Standing crop was estimated by clipping 0.25 m^2^ plots at 10 random sites in the pasture at the start of the study each year (Table 1).

All data were analyzed in R with the lmerTest (Kuznetsova et al., 2017) and lme4 (Bates et al., 2015) packages using a generalized linear mixed model. Each collared cow was considered an experimental unit. The 24-h period dataset was analyzed using a model that included 24-h period and year with a random intercept of individual cow. The hour-level dataset was analyzed using a model that included hour, 24-h period, and the hour by 24-h period interaction as fixed effects with a random intercept of individual cow. Means were separated with the emmeans package (Lenth, 2019) using the Tukey method. Statistical significance was accepted at *P* < 0.05.

## RESULTS

Daily activity data are presented in Table 2. Cows traveled 1.7 km further and grazed for 0.7 h less per day post-supplementation (*P* < 0.01). Resting time was similar pre- and post-supplementation (9.29 ± 0.25 h∙d^−1^; *P* = 0.07). There were no effects of year on daily activity (*P* > 0.30).

**Table 2.** Effect of three times weekly protein supplementation on the daily activity for the 24-h period pre-supplementation and 24-h period post-supplementation of beef cattle grazing native foothill rangeland between December and February over 2 yr in Norris, MT

	24-h*		SEM	Year^†^		SEM	*P*-value	
	Pre-supp.	Post-supp.		Year 1	Year 2		24-h	Year
Distance traveled, km∙d^−1^	5.18	6.90	0.69	6.07	6.02	0.82	<0.01	0.67
Grazing time, h∙d^−1^	12.90	12.20	0.21	12.70	12.30	0.28	<0.01	0.30
Resting time, h∙d^−1^	9.14	9.44	0.25	9.06	9.52	0.33	0.07	0.34

^*^24-h = 24-h period pre-supplementation (Pre-supp.) vs. 24-h period post-supplementation (Post-supp.)

^†^Year = Year 1 vs. Year 2.

**Figure 1.**
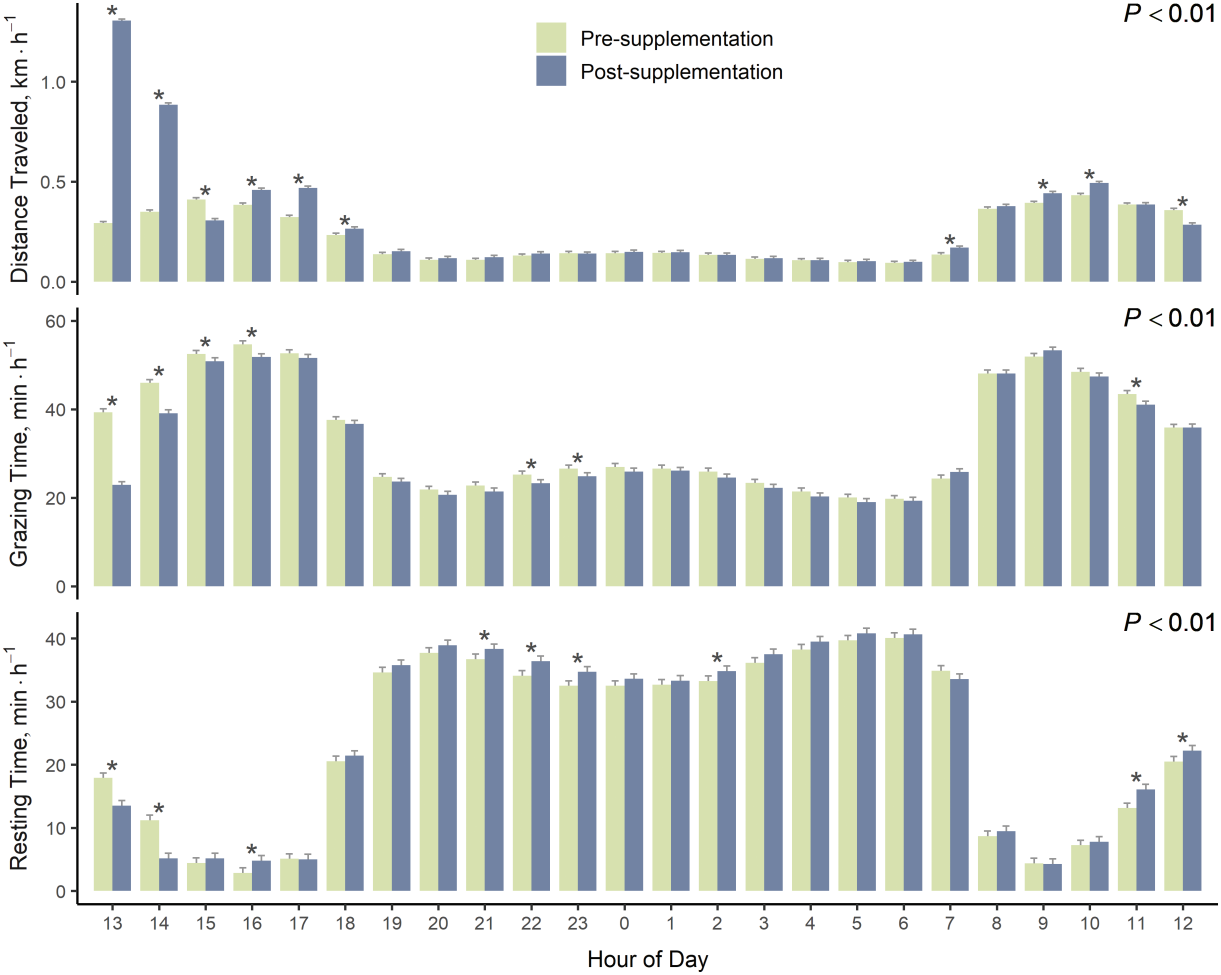
Hourly activity for the 24-h period pre-supplementation and 24-h period post-supplementation of beef cattle supplemented at 1300 h three times weekly, grazing native foothill rangeland between December and February over 2 yr in Norris, MT. Means are displayed with standard error bars. Asterisks above paired bars indicate a significant difference (*P* < 0.05) in the activity for that hour of the day. The *P*-value for each hour by 24-h supplementation period interaction was < 0.01.

Distance traveled, grazing time, and resting time displayed an hour by 24-h period interaction (*P* < 0.01; Fig. 1). Cows traveled further in the afternoon and morning hours post-supplementation (*P* < 0.02). Cows grazed less the afternoon and night post-supplementation (*P* < 0.05). Cows rested less the morning pre-supplementation and afternoon post-supplementation (*P* < 0.03). Cows also rested more at night post-supplementation (*P* < 0.05).

## DISCUSSION

Year 1 was coldest, received the most snow accumulation, and had over twice the amount of available forage compared to year 2. The mean daily temperature was less than 0 °C for 37 d in year 1 and 30 d in year 2. There was measurable snow coverage for 38 d in year 1 and 14 d in year 2. Despite the differences in weather conditions and forage availability, there was no main effect of year on daily distance traveled nor grazing and resting time.

Mean grazing time across years and supplementation periods was 12.5 h∙day^−1^, which is higher than 6–9 h, as has been reported for cattle grazing dormant vegetation (Adams et al., 1986; Prescott et al., 1994; Wyffels et al., 2019). This may due to differences in the classification of grazing, resting, and traveling during activity observation. Despite this difference, the pattern of grazing throughout the day was similar to previous studies, where most grazing occurs in the morning and afternoon (Ganskopp, 2001; Ganskopp and Bohnert, 2006; Schoenbaum et al., 2017). Therefore, relative differences between groups should remain consistent.

Most of the differences in distance traveled and grazing time between pre- and post-supplementation periods occurred in the hours immediately after the cows were supplemented. Ninety percent of the 1.7 km increase in distance traveled and 65% of the 0.7 h decrease in grazing time occurred within 5 h of supplementation. Similarly, others have noted cattle reduced grazing after they received supplement. Steers grazing Russian wildrye reduced grazing time for 4 h after they received supplement (Adams, 1985). Cows grazing winter rangeland in Idaho, receiving protein supplement once weekly, often reduced grazing time the day of or the day after supplement was delivered (Sprinkle et al., 2019). Conversely, in Montana, cows provided a protein supplement on alternate days increased grazing time in the 24-h period after supplementation (Dunn et al., 1988); however, the amount of supplement delivered in this study was 3.5 times less than in our study and was individually fed, which may have altered behavior. In our case, it is possible that feeding a larger quantity of a high-fiber protein supplement could have increased gut fill, reducing grazing post-supplementation.

Our results indicate that, for beef cows supplemented three times weekly, both daily activity and the hourly partitioning of activities differ pre- and post-supplementation. Most differences in daily activity are around the time when supplement is delivered; the remainder of the 24 h post-supplementation is largely the same as pre-supplementation. Therefore, infrequent protein supplementation as a tool to use low-quality forage and improve livestock distribution may result in minor changes to cow grazing behavior between supplemented and non-supplemented days. Continued research evaluating supplement delivery strategies that optimize use of low-quality forages on extensive rangeland environments are needed for western beef cattle production systems.



*Conflict of interest statement*. None declared.
